# Aflatoxin M1 in Milk and Dairy Products in Birjand, Iran: A Contribution to Human Health Risk Assessment

**DOI:** 10.1002/fsn3.70966

**Published:** 2025-09-14

**Authors:** Ayub Ebadi Fathabad, Mohammad Hasan Zarghi, Gholamreza Jahed Khaniki, Nabi Shariatifar, Negin Johari, Saeid Yousefi, Ramin Aslani

**Affiliations:** ^1^ Division of Food Safety and Hygiene, Department of Environmental Health Engineering, School of Public Health Tehran University of Medical Sciences Tehran Iran; ^2^ Department of Environmental Health Engineering, School of Public Health Tehran University of Medical Sciences Tehran Iran; ^3^ Department of Nutrition and Food Hygiene, School of Medicine Hamadan University of Medical Sciences Hamadan Iran; ^4^ Food and Drug Organization Birjand University of Medical Sciences Birjand Iran

**Keywords:** aflatoxin M1, health risk assessment, HPLC, ice cream, milk, yogurt

## Abstract

The objective of the current research was to examine the AFM1 contents in 150 milk and dairy product samples (including raw milk, UHT milk, yogurt, ice cream, and pasteurized milk) marketed in Birjand, Iran. The samples were analyzed utilizing high‐performance liquid chromatography with a fluorescence detector (HPLC‐FLD). The total mean of AFM1 in the raw milk, UHT milk, pasteurized milk, yogurt, and ice cream was 50.22 ± 12.39 ng/L, 12.17 ± 5.52 ng/L, 35.49 ± 11.07 ng/L, 13.03 ± 4.06 ng/kg, and 116.79 ± 40.35 ng/kg, respectively. The AFM1 contents of all samples were within the US FDA standard limits (500 ng/kg). The amounts of AFM1 in 16 samples of ice cream exceeded the limits set by the Iran National Standard Organization (INSO, 100 ng/kg). The contents of AFM1 in 41 samples exceeded the EU regulation (50 ng/kg). Furthermore, the health risks resulting from AFM1 occurrence in samples were evaluated using the Monte Carlo simulation. The 95th percentile value of the hazard index (HI) and the margin of exposure (MoE) in total samples for children was 9.028 and 2206, respectively, while those for adults were 1.934 and 10,295. The 95th percentile value of quantitative liver cancer risk (CR) in total samples for children and adults was 0.261 and 0.056 cancers/year/per 10^5^ individuals. The risk assessment results indicated the potential health risk posed by AFM1 in milk and dairy products. The implementation of appropriate management policies is recommended to prevent and control AFM1 contamination of food and feed chains.

## Graphical abstract

Aflatoxin M1 (AFM1) in milk and dairy products was determined using HPLC‐FLD, and the health risks of AFM1 in samples were also assessed. AFM1 in the samples varied from 6.28 to 196.25 ng/kg (mean = 43.18 ng/kg), with some samples containing AFM1 levels exceeding the standard limits. The risk assessments indicate that consuming milk and dairy products contaminated with AFM1 may pose significant health risks.

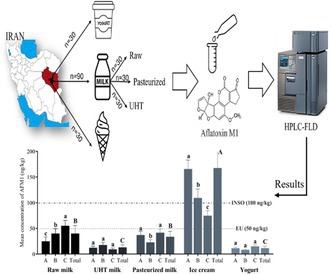

## 1 Introduction

Aflatoxins (AFs) are the most toxic mycotoxins typically produced by *Aspergillus* species, and can cause adverse health consequences, such as liver cancer and immune system disorders (El‐kest et al. 2015; Campagnollo et al. 2016; Abdali et al. 2020; Samimi et al. 2024). AFs commonly occur in agricultural products, particularly cereal grains, at the preharvest and postharvest stages, resulting in significant economic losses (Ul Hassan et al. 2018). Several factors contribute to fungal growth and AFs production, including high temperature and humidity, climate change, as well as storage duration and conditions (Iqbal et al. 2015).

Aflatoxin B1 (AFB1) is the most toxic among the 20 types of AF discovered so far, followed by G1, B2, and G2 (Ul Hassan et al. 2018; Samimi et al. 2024). AFB1 is easily absorbed in the gastrointestinal tract, and its main target is the liver. In the liver, AFB1 is metabolized and hydroxylated by cytochrome P450 (CYP450) enzymes, resulting in the formation of aflatoxin M1 (AFM1) and other metabolites. It has been reported that 12–14 h after AFB1‐contaminated food intake, AFM1 is detectable in milk, urine, and other body fluids of lactating animals (Atanda et al. 2007; Mazaheri et al. 2024). It is estimated that 0.3%–6.2% of ingested AFB1 is excreted in the form of AFM1 to milk; however, this ratio may differ according to animal species, individuals, exposure day, and milking stage (Creppy 2002; Atanda et al. 2007).

Milk is a basic component of a healthy diet since it is rich in nutrients (e.g., vitamins, minerals, protein, etc.). Moreover, several beneficial health effects are associated with milk consumption, including reducing the risk of cardiovascular disorders, regulating metabolic activities, and preventing osteoporosis (Hjartåker et al. 2002; Akbarieh et al. 2023). Currently, milk consumption is increasing worldwide, and the presence of AFM1 contamination poses a significant public health concern. Moreover, AFM1 binds to milk casein and remains relatively stable during storage and processing methods, such as sterilization and pasteurization. Therefore, its presence in various dairy products is expected (Vaz et al. 2020; Marimon Sibaja et al. 2022; Rasolipanah et al. 2022). Feeding lactating animals is accepted as a leading cause influencing milk safety and quality. There is a strong link between feeding lactating livestock with AFB1 contamination and the AFM1 level in milk. Milk produced by milking cows consuming AFB1 in their feed more than 70 μg per day is likely to exceed the standard limit of AFM1 specified by the European Union (EU, 50 ng/kg) (Hussein and Brasel 2001; Fallah 2010; Abdali et al. 2020). Although AFM1 is less hazardous compared to AFB1, it has been categorized as carcinogenic to humans (Group 1) and poses hepatotoxic effects (Vaz et al. 2020).

Different methods are employed to determine AFM1 in milk products, including thin‐layer chromatography (TLC) (Atanda et al. 2007; Fallah 2010), enzyme‐linked immunosorbent assay (ELISA) (Ertas et al. 2011; Abdali et al. 2020; Heshmati et al. 2020), high‐performance liquid chromatography (HPLC) with various detectors, such as fluorescence detector (FLD) (Rahimirad et al. 2014; Lee and Lee 2015) and mass spectrometry (MS) (Kos et al. 2016), direct analysis in real time with mass spectrometry (DART‐MS) (Busman et al. 2015), and liquid chromatography–tandem mass spectrometry (LC–MS/MS) (Wang et al. 2010). The ELISA technique is a rapid method, but its results may be false positives, and TLC has limitations in accurately quantifying AFs. Among them, HPLC is a reliable method, and HPLC‐FLD has been used in most studies (Lee and Lee 2015; Rasolipanah et al. 2022; Summa et al. 2025).

Recently, several research studies have been performed in various countries to determine AFM1 in dairy products, including Nigeria (Atanda et al. 2007), Turkey (Ertas et al. 2011), Korea (Lee and Lee 2015), Qatar (Ul Hassan et al. 2018), Iran (Moghaddam et al. 2019), Italy (Serraino et al. 2019), Yemen (Murshed 2020), Brazil (Conteçotto et al. 2021), Serbia (Milićević et al. 2021), Albania (Topi et al. 2022), China (Xiong et al. 2022), Latin America (Marimon Sibaja et al. 2022), Ethiopia (Tadesse et al. 2024), and Sri Lanka (Mudannayake et al. 2024). To the best of our knowledge, limited data are available on the AFM1 content in milk and dairy products in South Khorasan Province, Iran, and no comprehensive study has been carried out on this topic so far. Therefore, this study aimed to determine and assess health risks of AFM1 contents in milk (including raw milk, UHT milk, and pasteurized milk) and dairy products (including yogurt and ice cream) marketed in Birjand, Iran.

## 2 Materials and Methods

### 2.1 Study Area

**FIGURE 1:**
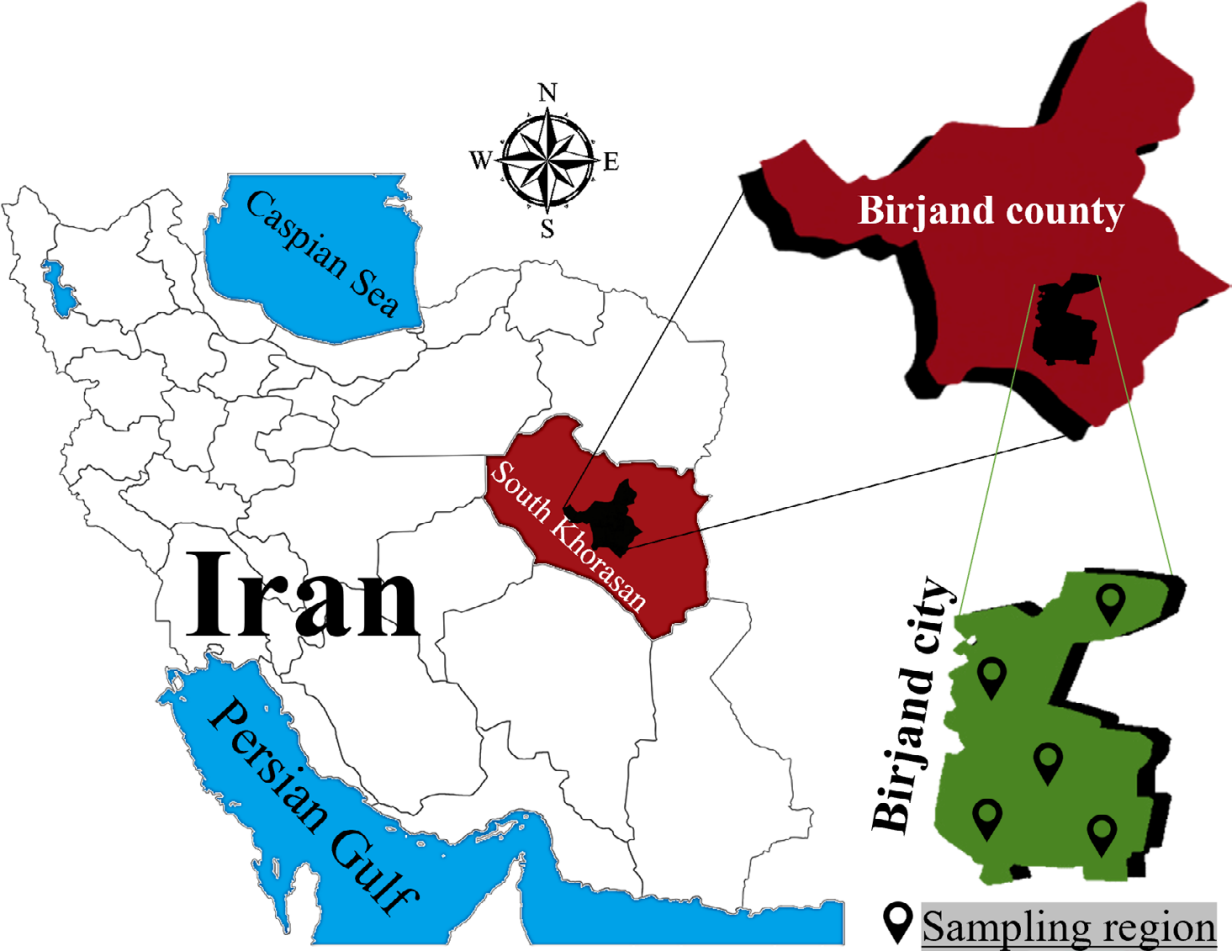
Sampling map of the study area.

The study was conducted in Birjand, the capital city of South Khorasan Province, Iran (Figure 1). Birjand is located at approximately 32.88° North latitude and 59.21° East longitude. It is the largest city in eastern Iran, covering an area of about 32.3 km^2^, with a population of over 200,000 (Saghafi 2018).

### 2.2 Sample Collection

In May 2024, a total of 150 samples of raw milk, pasteurized milk, UHT milk, ice cream, and yogurt (30 samples of each) were randomly collected from milk shops and supermarkets in five different locations in Birjand, Iran (Figure 1). All samples, except raw milk, were commercially produced and were from the three most popular brands. The milk and yogurt samples were plain and not flavored, while the ice cream was flavored with saffron. All samples were full‐fat, and each had a volume of 1 L or kg. The samples were kept at −18°C in the laboratory pending subsequent analysis.

### 2.3 Sample Preparation and Analysis

All chemicals used in this study were of analytical grade. AFM1 standard, acetonitrile, methanol, and other solvents were purchased from Sigma‐Aldrich (St. Louis, MO, USA). Initially, 20 mL of sample was poured into 80 mL distilled water and centrifuged for 6 min at 3000 × *g* (4°C); subsequently, the fatless portion was filtered using syringe filters (Whatman GD/X pore size 0.45 μm). Next, 20 mL of solution was passed (2–3 mL per min) through the immunoaffinity column (IAC), AflaStar Fit 3 (Romer Labs, Tulln, Austria), which was previously prepared with antibodies against AFM1 and kept at 25°C. The immunoaffinity column was rinsed twice with 10 mL of deionized water and dried. The bound AFM1 was eluted with acetonitrile and collected in a vial. The mobile phase comprises acetonitrile, methanol, and water (17:23:60 v/v/v) (ISIRI 2011; Shabansalmani and Movassaghghazani 2023; Behtarin and Movassaghghazani 2024). Finally, the samples (20 μL) were injected into an HPLC system (Alliance 2695, American Waters Company) with a fluorescence detector (FLR2475). A C18 HPLC column (25 cm × 5 mm × 5 μm) was utilized.

### 2.4 Method Validation

The analytical performance and validation for the determination of AFM1 in milk and dairy product samples including limit of detection (LOD, based on signal to noise ratio *n* = 3) and limit of quantification (LOQ, based on signal to noise ratio = 10), calibration curve equation and its linear range, coefficient of determination (*R* ^2^), and repeatability of results (RSD%, *n* = 3). The linearity was evaluated using AFM1 standard solutions at six concentration levels: 0.25, 0.5, 0.75, 1.0, 1.25, and 1.5 ppb. The y‐line equation was *y* = 431,245*x* − 11,130. The *R* ^2^ was 0.9979%, and the recovery rate was 109.1%. The RSD was 3.8%. The LOD and LOQ were 1.2 and 3.6 ng/kg, respectively.

### 2.5 Health Risk Assessment

#### 2.5.1 Estimation of Daily Intake (EDI)

The EDI values of AFM1 in milk and dairy samples for adults and children were computed utilizing the following formula:

(1) EDI=C×IR/BW

where C is the AFM1 level in samples, IR is the ingestion rate of different dairy products (milk = 0.14, yogurt = 0.073, ice cream = 0.016, and total milk and dairy products = 0.164 kg/day), and BW is body weight (adults = 70 and children = 15 kg) (Kiani et al. 2021; Shahsavari et al. 2022; Karimi et al. 2023).

#### 2.5.2 Hazard Index (HI)

The HI values of AFM1 in dairy products were obtained by Equation (2). The reference dose (RfD) value of AFM1 is considered 0.2 ng/kg bw/day (Rahmani et al. 2018; Fakhri et al. 2019; Mohammadi et al. 2021; Massahi et al. 2024). Since AFM1 is a genotoxic and carcinogenic compound, HI is used for comparative purposes and not as a basis for risk characterization.

(2) HI=EDI/RfD

#### 2.5.3 Margin of Exposure (MoE)

The margin of exposure (MoE) represents the ratio of a toxicological reference point to a dose that provokes a slight but discernible response (Authority 2005; Dominguez et al. 2024). The BMDL_10_ of AFM1 is considered 4 μg/kg bw/day (Milićević et al. 2021; Ghaffarian‐Bahraman et al. 2023). The MoE of AFM1 was computed via the following equation:

(3) MoE=BMDL10/EDI

#### 2.5.4 Quantitative Liver Cancer Risk (CR)

AFM1 triggers liver cancer with 10% of the potency of AFB1 (Joint et al. 1998). Additionally, the incidence of liver cancer caused by AFs is synergistically amplified by HBV infection. Hence, the liver carcinogenic potency of AFM1 for people with positive (HBsAg^+^) and negative (HBsAg^−^) HBV infections is considered 0.03 and 0.001 cancers/year per 10^5^ individuals per ng/kg bw/day. The HBsAg^+^ prevalence rate in the Iranian population is 2.2% (Guo et al. 2013; Conteçotto et al. 2021; Samimi et al. 2024). The quantitative liver cancer risk associated with AFM1 in milk and dairy products was evaluated via the following formulas:

(4) $$\text{Pcancer}=\left(0.001\times \%\text{HBsAg}-\right)+\left(0.03\times \%\text{HBsAg}+\right)$$

(5) CR=EDI×Pcancer

### 2.6 Statistical Analysis

Data analysis was conducted in IBM SPSS Statistics version 27 (IBM Corp, Armonk, NY, USA), using the Kruskal–Wallis test (*p* < 0.05). Oracle Crystal Ball version 11.1.2.4.850 (Oracle Corporation, Redwood Shores, CA, USA) was utilized to execute the Monte Carlo simulation. The 95th percentile probability risk values were considered significant risks, and the trial numbers were 10,000 iterations.

## 3 Results and Discussion

### 3.1 Occurrence of AFM 1 in Milk and Dairy Products

**FIGURE 2:**
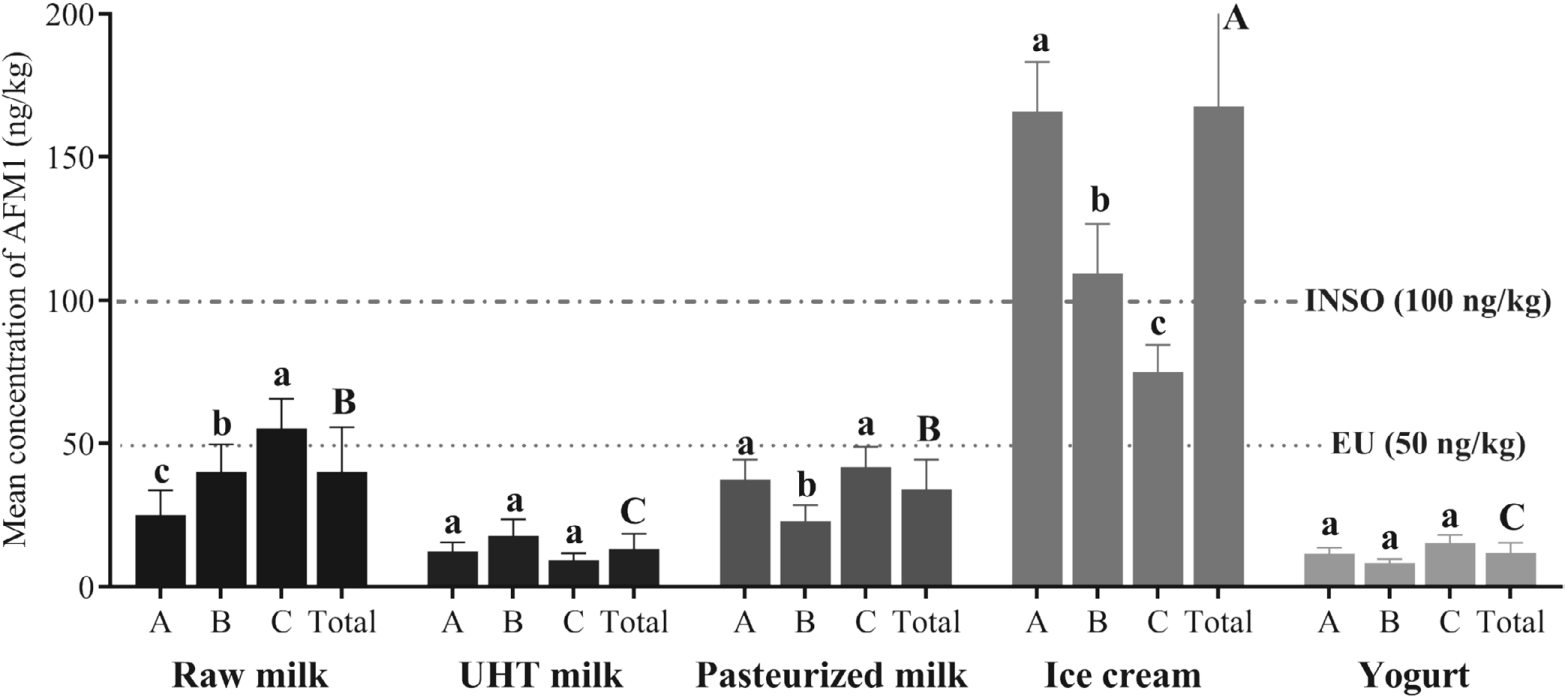
The mean concentration of AFM1 in three brands (A, B, and C) of milk and dairy product samples, and the total AFM1 concentration in each sample type. Different small letters indicate statistically significant differences (*p* < 0.05) in AFM1 concentration in different brands of each product type. Different capital letters indicate statistically significant differences (*p* < 0.05) in total AFM1 concentration in product types. Dashed lines represent the maximum permissible limits set by the European Union (EU, 50 ng/kg) and the Iranian National Standard Organization (INSO, 100 ng/kg).

According to the high consumption of dairy products, the presence of AFM1 can pose risks to consumers health. Table 1 provides the content of AFM1 measured in the dairy samples collected from Birjand, Iran. AFM1 was detected in 100% of the studied samples. Heidari et al. (2024) found that 85.55% of 180 traditional dairy product samples in Iran were positive for AFM1 (Heidari et al. 2024). The average concentration of AFM1 in the total samples was 43.18 ± 43.40 ng/kg and ranged from 6.28 to 196.25 ng/kg. As shown in Figure 2, the increasing order of AFM1 means concentrations in different samples were as follows: yogurt < UHT milk < pasteurized milk < raw milk < ice cream. AFM1 levels in all the samples were within the standard limits recommended by the United States Food and Drug Administration (US FDA) (500 ng/kg) (Alimentarius 1995; Food and Administration 2005). AFM1 contents in 16 samples of ice cream were above the limits set by the Iran National Standard Organization (INSO No. 5925) (100 ng/kg) (Standards and Iran 2002). AFM1 amounts in 9 samples of raw milk, 2 samples of pasteurized milk, and 30 samples of ice cream were higher than the limit established in the European Union (EU) (50 ng/kg) (Union 2006). These results align with some previous research, which reported that the AFM1 amounts were above the standard limits (Ul Hassan et al. 2018; Abdali et al. 2020). Summa et al. (2025) found that AFM1 levels in 70 of 1017 milk samples exceeded the EU standard (Summa et al. 2025). In contrast, Xiong et al. (2022) found that the AFM1 in 288 pasteurized milk, 108 UHT milk, and 329 yogurt samples was less than the Chinese standard (500 ng/L) (Xiong et al. 2022), which can be linked to the good agricultural and storage practices employed in the feed chain (Fallah 2010).

**TABLE 1:** Concentration of AFM1 in milk and dairy product samples.

Sample	Brands	No. samples	No. samples above standard limits			AFM1 concentrations (ng/L, ng/kg)				
			EU	INSO	FDA	Min	Max	Median	Mean	SD
Raw milk	A	10	0	0	0	12.76	40.02	24.95	25.08	8.62
	B	10	2	0	0	26.49	56.74	39.05	40.14	9.60
	C	10	7	0	0	38.52	71.41	55.03	55.27^d^	10.39
	Total	30	9	0	0	12.76	71.41	39.32	40.16^B^	15.60
UHT milk	A	10	0	0	0	8.74	17.36	12.50	12.36	3.10
	B	10	0	0	0	9.53	27.39	17.15	17.75	5.81
	C	10	0	0	0	6.28	13.91	8.65	9.38	2.35
	Total	30	0	0	0	6.28	27.39	11.76	13.16^C^	5.32
Pasteurized milk	A	10	0	0	0	27.89	47.47	38.40	37.34	7.05
	B	10	0	0	0	17.26	33.54	20.92	22.95	5.56
	C	10	2	0	0	30.11	52.63	41.00	41.77	7.10
	Total	30	2	0	0	17.26	52.63	34.27	34.02^B^	10.40
Ice cream	A	10	10	10	0	135.58	196.25	165.50	165.82^a^	17.45
	B	10	10	6	0	82.92	136.70	161.15	109.46^b^	17.24
	C	10	10	0	0	59.23	89.31	77.15	75.11^c^	9.36
	Total	30	30	16	0	59.23	196.25	161.15	167.79^A^	40.35
Yogurt	A	10	0	0	0	8.76	14.62	11.82	11.63	2.00
	B	10	0	0	0	6.33	10.95	8.20	8.41	1.36
	C	10	0	0	0	11.92	21.26	15.2	15.34	2.79
	Total	30	0	0	0	6.33	21.26	11.80	11.79^C^	3.54
Total	—	150	41	16	0	6.28	196.25	27.35	43.18	43.40

*Note:* Different small letters in the column indicate statistically significant differences (*p* < 0.05) in different brands, and Different capital letters in the column indicate statistically significant differences (*p* < 0.05) in dairy product types.

According to Table 1, AFM1 concentration in raw milk varied from 12.76 to 71.41 ng/L, and the mean value was 40.16 ± 15.60 ng/L. The average amounts of AFM1 in the different brands of raw milk samples decreased as follows: C (55.27 ± 10.39 ng/L) > B (40.14 ± 9.06 ng/L) > A (25.08 ± 8.62 ng/L). The variation in AFM1 levels among different milk and dairy product brands is influenced by multiple factors, including the source and quality of raw milk, the processing methods employed, and the effectiveness of food safety management and quality control systems implemented by producers (Abdali et al. 2020). There were significant differences between raw milk, yogurt, and UHT milk (*p* < 0.05), but there were no significant differences between raw milk and pasteurized milk (*p* > 0.05). Ertas et al. (2011) detected AFM1 in 86% of 50 raw milk samples from Turkey, ranging from 1 to 30 ng/L (Ertas et al. 2011). Behtarin and Movassaghghazani (2024) found that the average amount of AFM1 in 8 raw milk samples was 36.22 ± 2.45 ng/L (ranging from 28.30 to 46.60 ng/L). Additionally, the average value of AFM1 in pasteurized milk was 28.41 ± 2.36 ng/L, and in UHT milk was 21.16 ± 1.54 ng/L (Behtarin and Movassaghghazani 2024). Poor agriculture and animal feed storage conditions cause fungi contamination and the generation of AFB1. The AFM1 in milk is affected by seasonal changes, country development status, and contamination of feed with AFB1 (Rahimirad et al. 2014; Ul Hassan et al. 2018). For example, AFM1 in raw milk produced in the summer was lower than that in the winter, particularly because animals were fed lower‐quality feed in the winter (Fallah 2010). In a study, the average amounts of AFM1 in raw milk in summer and winter were 49 and 14 ng/L, respectively (Hasninia et al. 2022). Djekic et al. (2020) reported that the mean level of AFM1 in raw milk samples collected in winter, spring, summer, and autumn was 0.057, 0.081, 0.050, and 0.220 μg/kg, respectively. They also stated that since AFs contamination varies between harvests and is affected by agricultural practices and seasonal changes, it is crucial to improve farming practices to reduce the occurrence of AFB1 (Djekic et al. 2020). The presence of high contents of AFM1 in raw milk results in the occurrence of AFM1 in other products. Therefore, it is extremely critical to produce milk with low levels of AFM1 contamination to ensure dairy product safety (Atanda et al. 2007).

The mean concentration of AFM1 in the UHT milk was 13.16 ± 5.32 ng/L and ranged from 6.28 to 27.39 ng/L. The descending order of mean AFM1 concentrations in different brands of UHT milk was as follows: B (17.75 ± 5.81 ng/L) > A (12.36 ± 3.10 ng/L) > C (9.38 ± 2.35 ng/L). AFM1 contents determined in UHT milk in our study were less than those recorded by Lee and Lee (2015) (Lee and Lee 2015). Xiong et al. (2022) reported that AFM1 in UHT milk ranged from 5.1 to 46.6 ng/L (Xiong et al. 2022). In another study, AFM1 was measured using HPLC‐FLD, and the mean level in UHT milk was 70 ± 8 ng/L (Mwakosya and Mugula 2021). Authors in Turkey detected the mean concentration of AFM1 in UHT milk at 20.29 ng/L (Turkoglu and Keyvan 2019).

The mean value of AFM1 in pasteurized milk was 34.02 ± 10.40 ng/L (ranging from 17.26 to 52.63 ng/L). The average levels of AFM1 in the different brands of pasteurized milk samples decreased in the following order: C (41.77 ± 7.10 ng/L) > A (37.34 ± 7.05 ng/L) > B (22.95 ± 5.56 ng/L). The average amount of AFM1 in pasteurized milk in our study was higher than that found by Madali et al. (2018) and Lee and Lee (2015), as well as lower than Conteçotto et al. (2021) findings (Conteçotto et al. 2021). Fallah (2010) reported that the contents of AFM1 in pasteurized milk varied from 0.013 to 0.250 μg/L and that 36.2% of samples had AFM1 contents exceeding standard limits (Fallah 2010). In another study, AFM1 in pasteurized milk was 1.237 ± 1.317 μg/L (Tadesse et al. 2024).

In the current research, the rank order of AFM1 levels in milk samples was raw milk > pasteurized milk > UHT milk. There was no significant difference in AFM1 content between raw milk and pasteurized milk (*p* > 0.05), which is in agreement with other studies (Sefidgar et al. 2011; Rahimirad et al. 2014). The AFM1 contents in UHT milk and pasteurized milk were significantly different (*p* < 0.05). The difference in AFM1 levels in different milk samples is likely due to heat processing conditions (Xiong et al. 2022). Omeiza et al. (2018) concluded that the sterilization and pasteurization processes of milk decreased the AFM1 concentration by 58.8% and 7.1%, respectively (Omeiza et al. 2018). However, AFs are extremely resistant to heat, and complete degradation is reached at temperatures varying from 237°C to 306°C (Ul Hassan et al. 2018).

The average level of AFM1 in the yogurt was 11.79 ± 3.54 ng/kg and varied from 6.33 to 21.26 ng/kg. The highest and lowest average AFM1 levels were found in brands C (15.34 ± 2.79 ng/kg) and B (8.41 ± 1.36 ng/kg), respectively. There were significant differences between yogurt and various dairy products (*p* < 0.05), except between yogurt and UHT milk (*p* > 0.05). AFM1 concentrations higher than those observed in the present research can be found in research conducted by (Ul Hassan et al. 2018), Lee and Lee (2015) (Lee and Lee 2015), and Fallah (2010). Another study found that the mean level of AFM1 in yogurt from China was 20.7 ± 9.7 ng/kg (ranging from 10.0 to 66.7 ng/kg) (Xiong et al. 2022). Sumon et al. (2021) examined AFM1 in dairy products using the ELISA method and reported that the average amount of AFM1 in yogurt was 16.9 ± 13.6 ng/kg (Sumon et al. 2021). In the study by Murshed (2020), the AFM1 content in yogurt ranged from 0.053 to 893 μg/kg (Murshed 2020). In the current study, yogurt contained the lowest AFM1 levels among the different dairy products. In accordance with our findings, Rahimirad et al. (2014) found the minimum amount of AFM1 in yogurt among different dairy products (raw milk, pasteurized milk, cream, and cheese) (Sefidgar et al. 2011; Rahimirad et al. 2014). This can be explained by the fact that yogurt is a fermented dairy product with lactic acid bacteria (LAB), and numerous reports have shown the ability of LAB to decrease the amount of AFM1 during fermentation (El Khoury et al. 2011; Barukčić et al. 2018). In contrast, some research has indicated that AFM1 concentrations in yogurt are not reduced during fermentation (Iha et al. 2013). On the other hand, lactic acid in dairy products may react with AFM1 and produce AFM2, which is a less toxic metabolite than AFM1 (Shukla et al. 2002; Lee and Lee 2015).

AFM1 mean values in the ice cream were 167.79 ± 40.35 ng/kg (ranging from 59.23 to 196.25 ng/kg). The average amount of AFM1 in the different brands of ice cream samples decreased as follows: A (165.82 ± 17.45 ng/kg) > B (109.46 ± 17.24 ng/kg) > C (75.11 ± 9.36 ng/kg). AFM1 concentration in the ice cream was significantly higher than in the other products (*p* < 0.05). This may be attributed to the processing of the ice cream. The condensed milk or milk powder used for ice cream production can cause an increase in AFM1 levels in the final product. Rahimi (2014) reported that 56.7% of 60 ice cream samples were positive for AFM1, and the mean value of AFM1 was 65.1 ± 31.4 ng/kg (ranging from 14.9 to 147.4 ng/kg) (Rahimi 2014). AFM1 levels in ice cream detected in the present research were more than those found by Lee and Lee (2015) (Lee and Lee 2015). Abdali et al. (2020) found that the AFM1 contents in 22 ice creams from Shiraz, Iran, varied from 0.3 to 71.1 ng/kg (mean = 26.88 ng/kg) (Abdali et al. 2020). In another study by Rasolipanah et al. (2022), the average amount of AFM1 in ice cream was 29.79 ng/kg, and the maximum concentration was 87.8 ng/kg (Rasolipanah et al. 2022). Fallah (2010) detected AFM1 in 25 samples of ice cream with a mean amount of 0.041 μg/kg, which was 27.7% of samples exceeding standard levels (Fallah 2010).

### 3.2 Health Risk Assessment

Table 2 indicates the health risk assessment results of AFM1 in dairy products for Iranian children and adults. The EDI, HI, MoE, and CR values of AFM1 in total milk and dairy products for adults were 0.387 ng/kg bw/day, 1.934, 10,295, and 0.056 cancers/year/10^5^ individuals, respectively, while these values for children were 1.806 ng/kg bw/day, 9.028, 2206, and 0.261 cancers/year/10^5^ individuals, respectively. The 95th percentile EDI values of AFM1 in raw milk, UHT milk, pasteurized milk, ice cream, and yogurt for adults were 0.135, 0.048, 0.101, 0.042, and 0.019 ng/kg bw/day, respectively, and these values for children were 0.628, 0.226, 0.471, 0.197, and 0.090 ng/kg bw/day, respectively. Guo et al. (2013) found that the 95th percentile EDI value in milk products from China ranged from 0.033 to 0.038 ng/kg bw/day (Guo et al. 2013). In another study, the EDI values of AFM1 in raw milk, pasteurized milk, UHT milk, and yogurt for children were 0.46, 0.37, 0.27, and 0.13 ng/kg bw/day, respectively (Behtarin and Movassaghghazani 2024).

**TABLE 2:** EDI, HI, MoE, and CR values of AFM1 exposure through milk and dairy product consumption by adults and children.

Samples	Percentiles	Risk scenario							
		EDI (ng/kg bw/day)		HI		MoE		CR	
		Adults	Children	Adults	Children	Adults	Children	Adults	Children
Raw milk	25%	0.056	0.259	0.278	1.297	124,194	26,613	0.008	0.038
	50%	0.079	0.368	0.395	1.842	71,967	15,421	0.011	0.053
	75%	0.104	0.487	0.522	2.434	50,664	10,856	0.015	0.070
	95%	0.135	0.628	0.673	3.139	29,727	6370	0.019	0.091
UHT milk	25%	0.018	0.085	0.091	0.424	287,850	61,682	0.003	0.012
	50%	0.024	0.111	0.119	0.556	220,177	47,180	0.003	0.016
	75%	0.032	0.149	0.159	0.743	167,859	35,969	0.005	0.022
	95%	0.048	0.226	0.242	1.128	82,680	17,717	0.007	0.033
Pasteurized milk	25%	0.051	0.239	0.256	1.196	116,392	24,941	0.007	0.035
	50%	0.069	0.320	0.343	1.599	78,020	16.718	0.010	0.046
	75%	0.085	0.399	0.427	1.993	58,339	12,501	0.012	0.058
	95%	0.101	0.471	0.504	2.353	39,665	8499	0.015	0.068
Ice cream	25%	0.018	0.085	0.091	0.425	282,750	60,589	0.003	0.012
	50%	0.024	0.113	0.121	0.565	219,572	47,051	0.004	0.016
	75%	0.032	0.149	0.160	0.747	165,037	35,365	0.005	0.022
	95%	0.042	0.197	0.212	0.987	94,466	20,242	0.006	0.029
Yogurt	25%	0.009	0.044	0.047	0.220	550,461	117,956	0.001	0.006
	50%	0.012	0.055	0.059	0.276	424,222	90,905	0.002	0.008
	75%	0.015	0.069	0.074	0.343	338,619	72,561	0.002	0.010
	95%	0.019	0.090	0.096	0.449	207,718	44,511	0.003	0.013
Total	25%	0.032	0.148	0.159	0.742	213,707	45,794	0.005	0.021
	50%	0.057	0.268	0.287	1.341	125,730	26,942	0.008	0.039
	75%	0.122	0.567	0.608	2.836	69,569	14,907	0.018	0.082
	95%	0.387	1.806	1.934	9.028	10,295	2206	0.056	0.261

The 95th percentile HI values of AFM1 in raw milk, UHT milk, pasteurized milk, ice cream, and yogurt for adults were 0.673, 0.242, 0.504, 0.212, and 0.096, respectively, while these values for children were 3.139, 1.128, 2.353, 0.987, and 0.449, respectively. HI values above one indicate adverse health outcomes, and vice versa. HI values of AFM1 in raw, UHT, and pasteurized milk samples for children were above one and unacceptable. In alignment with our findings, the HI values of raw, pasteurized, and UHT milk for children reported by Behtarin and Movassaghghazani (2024) were 2.22, 1.74, and 1.30, respectively, which were more than one and unacceptable (Behtarin and Movassaghghazani 2024). Authors in Ethiopia found that the HI value of AFM1 in pasteurized milk was 7.5 for adults and 26 for children (Tadesse et al. 2024). In contrast, Serraino et al. (2019) state that the HI values of AFM1 in raw milk from Italy for both children and adults were below one and considered acceptable (Serraino et al. 2019). In another study, the HI values of AFM1 in milk were less than one for males, females, and children (Ghaffarian‐Bahraman et al. 2023).

The 95th percentile MoE values in raw milk, UHT milk, pasteurized milk, ice cream, and yogurt for adults were 29,727, 82,680, 39,665, 94,466, and 207,718, respectively. The 95th percentile MoE values in raw milk, UHT milk, pasteurized milk, ice cream, and yogurt for children were 6370, 17,717, 8499, 20,242, and 44,511, respectively. MoE values below 10,000 are regarded as a public health concern (Samimi et al. 2024). Ghaffarian‐Bahraman et al. (2023) indicate that the MoE values of AFM1 in milk in southeastern Iran for males, females, and children were 14,833, 13,044, and 6719, respectively (Ghaffarian‐Bahraman et al. 2023). In another study, the average MoE values of AFM1 in different dairy samples in Serbia for 1–9‐year‐old children ranged from 123,316 to 194,657 (Milićević et al. 2021). The MoE value of AFM1 from pasteurized milk for adults was 380 in the study by Tadesse et al. (2024) (Tadesse et al. 2024). Additionally, a study concluded that the MoE values of AFM1 for children ranged from 728 to 239 (Conteçotto et al. 2021).

The 95th percentile CR values of AFM1 in raw milk, UHT milk, pasteurized milk, ice cream, and yogurt for adults were 0.019, 0.007, 0.015, 0.006, and 0.003 cancers/year/per 10^5^ individuals, respectively, while these values for children were 0.091, 0.033, 0.068, 0.029, and 0.013 cancers/year/10^5^ individuals, respectively. These findings show that children are prone to a considerably higher risk of liver cancer than adults. Conteçotto et al. (2021) reported that the CR values of AFM1 exposure in dairy products by 0 to < 6‐year‐old children ranged from 0.0027 to 0.0029 cancers/year/per 10^5^ individuals (Conteçotto et al. 2021). Guo et al. (2013) found 24.6 cancers/year/per 10^8^ individuals resulting from AFM1 in milk in the general Chinese population (Guo et al. 2013). The mean CR reported by Tadesse et al. (2024) was 0.007978 cancers/year/per 10^5^ individuals (Tadesse et al. 2024). In another study, the median CR values of AFM1 from milk consumption were 0.0006, 0.0007, and 0.0013 cancers/year/per 10^5^ individuals for males, females, and children, respectively (Ghaffarian‐Bahraman et al. 2023). The 95th percentile mean CR value of AFM1 in pasteurized and UHT milk for 1–3‐year‐old female toddlers reported by Milićević et al. (2021) was 0.00089 cancers/year/per 10^5^ individuals (Milićević et al. 2021).

## 4 Conclusion

Food safety has received substantial attention in recent decades. AFM1 in dairy products has raised considerable concerns worldwide, so the objective of this research was to examine AFM1 in milk and dairy products in Birjand, Iran. AFM1 in the samples varied from 6.28 to 196.25 ng/kg (average = 43.18 ng/kg). Some samples contained AFM1 amounts exceeding the maximum standard limits. The maximum and minimum mean concentrations were found in ice cream (167.79 ± 40.35 ng/kg) and yogurt (11.79 ± 3.54 ng/kg), respectively. The 95th percentile HI, MoE, and CR values of exposure to AFM1 in total samples for adults were 1.934, 10,295, and 0.056, respectively. For children, these values were 9.028, 2206, and 0.261, respectively. The risk assessment findings indicate potential health risks, especially for children. Considering the inherent thermal resistance characteristics of AFM1, preventing contamination of livestock feed with AFB1 is a key factor in controlling AFM1 levels. Good manufacturing practices, good agricultural practices, prevention of fungal growth in feedstuffs, and effective management policies in the food and feed chain can contribute to the production of safer milk by lactating animals. The utilization of chemical, physical, or biological treatment methods to diminish AFs in the food and feed chains is also recommended.

## Author Contributions

**Ayub Ebadi Fathabad:** formal analysis, data curation, investigation, writing – original draft, writing – review and editing. **Mohammad Hasan Zarghi:** formal analysis, investigation, writing – original draft. **Gholamreza Jahed Khaniki:** project administration, formal analysis, data curation, writing – review and editing. **Nabi Shariatifar:** project administration, formal analysis, data curation, writing – review and editing. **Negin Johari:** writing – original draft, investigation. **Saeid Yousefi:** literature search, data curation, resources, methodology. **Ramin Aslani:** supervision, conceptualization, project administration, writing – review and editing.

## Conflicts of Interest

The authors declare no conflicts of interest.

## Data Availability

The data that support the findings of this study are available on request from the corresponding author.
